# Protein *N*-glycosylation in eukaryotic microalgae and its impact on the production of nuclear expressed biopharmaceuticals

**DOI:** 10.3389/fpls.2014.00359

**Published:** 2014-07-28

**Authors:** Elodie Mathieu-Rivet, Marie-Christine Kiefer-Meyer, Gaëtan Vanier, Clément Ovide, Carole Burel, Patrice Lerouge, Muriel Bardor

**Affiliations:** ^1^Laboratoire Glyco-MEV, Faculté des Sciences et Techniques, UPRES EA 4358, Normandie Université, IRIB, VASIMont-Saint-Aignan, France; ^2^Institut Universitaire de FranceParis, France

**Keywords:** microalgae, biopharmaceuticals, glycosylation pathway, glycan, *Chlamydomonas reinhardtii*, *Phaeodactylum tricornutum*, endoplasmic reticulum, Golgi apparatus

## Abstract

Microalgae are currently used for the production of food compounds. Recently, few microalgae species have been investigated as potential biofactories for the production of biopharmaceuticals. Indeed in this context, microalgae are cheap, classified as Generally Recognized As Safe (GRAS) organisms and can be grown easily. However, problems remain to be solved before any industrial production of microalgae-made biopharmaceuticals. Among them, post-translational modifications of the proteins need to be considered. Especially, *N*-glycosylation acquired by the secreted recombinant proteins is of major concern since most of the biopharmaceuticals are *N*-glycosylated and it is well recognized that glycosylation represent one of their critical quality attribute. Therefore, the evaluation of microalgae as alternative cell factory for biopharmaceutical productions thus requires to investigate their *N*-glycosylation capability in order to determine to what extend it differs from their human counterpart and to determine appropriate strategies for remodeling the microalgae glycosylation into human-compatible oligosaccharides. Here, we review the secreted recombinant proteins which have been successfully produced in microalgae. We also report on recent bioinformatics and biochemical data concerning the structure of glycans *N*-linked to proteins from various microalgae phyla and comment the consequences on the glycan engineering strategies that may be necessary to render those microalgae-made biopharmaceuticals compatible with human therapy.

## Introduction

Nowadays, biopharmaceuticals on the market consists of 200 products yielding overall global revenue greater than US$100 billion (Walsh, 2010). Over the past 5 years, 140 biopharmaceuticals were approved in the European Union (EU) and the United States (US) markets (Walsh, 2010). These biopharmaceuticals are produced in various systems ranging from bacteria to mammalian cell cultures (Wong, 2005; Demain and Vaishnav, 2009; Huang et al., 2012). Among those, the Chinese Hamster Ovary (CHO) cells are currently the predominant industrial cell lines used for producing those drugs (Hossler et al., 2009), covering about 50% of the market (Demain and Vaishnav, 2009). However, the constant increasing needs for large amount of such therapeutic proteins, their high production cost in conventional expression systems and complicating factors related to potential virus contamination have driven scientists to explore new alternative production systems. In this context, various plant expression systems have emerged, including whole plants (Nicotiana, Alfalfa, Maize for examples) and *in vitro* culture systems such as plant cell suspensions (*Nicotiana tabaccum, Lemna minor, Physcomitrella patens*) and hairy roots (Stoger et al., 2005; Drake et al., 2009; Colgan et al., 2010; Decker and Reski, 2012; Parsons et al., 2012; Xu et al., 2012; Schillberg et al., 2013; Twyman et al., 2013; Buyel and Fischer, 2014). Several plant-made biopharmaceuticals have been successfully produced (De Muynck et al., 2010). This includes the Cerezyme's biosimilar (glucocerebrosidase) which has been produced in carrot cells (Shaaltiel et al., 2007) and was approved on May 2012 by the US Food and Drug Administration (Maxmen, 2012). Despite those successes, there is an increasing interest to use microalgae for biopharmaceutical production. Microalgae are unicellular photosynthetic organisms which encompass between 40,000 and probably several billion species (Cadoret and Bernard, 2008; Mata et al., 2010). As plants, microalgae are classified in Generally Recognized As Safe (GRAS) organisms. Moreover, they are cheap and easy to grow, making them potentially attractive cell factories for the large-scale production of recombinant proteins.

To date, microalgae have been mainly used for the production of food compounds or high-value added compounds like carotenoids (Spolaore et al., 2006; Sasso et al., 2012). In addition, as photosynthetic organisms, microalgae are very efficient in converting sunlight into chemical energy, making them attractive for the production of carbohydrates, lipids, and hydrogen. Therefore, algal biomass represents a great potential for generating new sources of bioenergy such as biofuels (Beer et al., 2009; Lam and Lee, 2012; Merchant et al., 2012) and biomaterials (Hempel et al., 2011a). Several microalgae species have also been evaluated for their potential to express recombinant proteins. Among them, the model Chlorophyceae *Chlamydomonas reinhardtii* is currently the most investigated one for such a biotechnological application, due to the availability of genomic data and the existence of a powerful molecular toolkit including vectors allowing nuclear or chloroplastic transformation (Merchant et al., 2007; Harris, 2009). Additionally, the diatom *Phaeodactylum tricornutum* is also considered as an emerging system for such an application (Hempel et al., 2011b; Hempel and Maier, 2012) as it can be genetically modified and grown quite easily. Other species are also good candidates for large-scale production of recombinant proteins, especially due to their ease to obtain algal biomass and their growth rate. For example, a marine green microalga from the class of the Chlorophyceae, *Dunaliella salina* (Geng et al., 2003), but also species from the genus *Chlorella* [i.e., *Chlorella vulgaris*, recently renamed *Coccomyxa* sp. C-169 *subellipsoidea* (Blanc et al., 2010), *Chlorella ellipsoidea*], which belongs to the Trebouxiophyceae, are promising bio-factories for large-scale production of high-value added proteins (Hawkins and Nakamura, 1999; Chen et al., 2001; Kim et al., 2002; Bai et al., 2013). However, molecular biology tools remain poorly developed for most of those microalgae even if genomic data became available (http://genome.jgi.doe.gov/; http://www.phytozome.net/; http://www.ncbi.nlm.nih.gov/genome).

Whatever the considered species, many problems have still to be solved before any industrial production and commercialization of microalgae-made biopharmaceuticals. Among them, increasing the yield and secretion of recombinant protein represents a crucial issue to make these organisms competitive with traditionally used expression systems such as the CHO cell lines. Furthermore, post-translational modifications need to be considered. Especially, *N*-glycosylation acquired by the secreted recombinant proteins is crucial for biopharmaceuticals since more than one third of the approved ones are glycosylated (Gomord et al., 2010) and it represents a critical quality attribute for them (Lingg et al., 2012). Indeed, the presence and structures of the *N*-glycans are required for their biological activity, stability and half-life (Lingg et al., 2012). The evaluation of microalgae as alternative cell factory for biopharmaceutical production thus requires investigating their *N*-glycosylation capability in order to determine to what extend their *N*-glycans differ from their human counterpart. In this paper, we report on bio-informatic and biochemical data concerning the structures of glycan *N*-linked to endogenous proteins from various microalgae phyla. Based on these recent findings, strategies for the engineering of the glycosylation pathways in these new expression systems are proposed to obtain microalgae-made biopharmaceuticals that would carry oligosaccharides compatibles with human therapies.

## Microalgae as alternative systems for production of recombinant proteins

### Available tools for nuclear transformation in microalgae

#### Chlamydomonas reinhardtii

Currently, most of the studies reporting the expression of recombinant proteins in microalgae have been performed in the green alga *Chlamydomonas reinhardtii*. The majority of such productions has been targeted to the chloroplast. This concerned biopharmaceuticals such as the single-chain antibody directed against the glycoprotein D of the herpes simplex virus (Mayfield et al., 2003), but also the heavy and light chains of the antibody 83K7C, derived from a human IgG1 (Tran et al., 2009), human erythropoietin, domains 10 and 14 of human fibronectin, interferon β1, proinsulin, vascular endothelial growth factor (VEGF), high mobility group protein B1(HMGB1) (Rasala et al., 2010) and two different immunotoxin proteins (Tran et al., 2012). Recently, an attenuated form of the E7 oncoprotein of the human papillomavirus (HPV) has also been produced in the same organelle (Demurtas et al., 2013). Indeed, this strategy is the most promising one to get high protein yields since *C. reinhardtii* possesses a large single chloroplast, representing about 40% of the total cell volume, and the proteins expressed in this organelle have been shown to represent 2–20% of total soluble proteins (Rasala and Mayfield, 2011). Moreover, three proteins have been shown to be expressed at levels which are sufficient for commercial production (Rasala et al., 2010). In contrast, secreted proteins produced from nuclear transformation in *C. reinhardtii* generally failed to accumulate to an equivalent level as the one observed in chloroplasts (Fuhrmann et al., 1999; Schroda et al., 2000; Specht et al., 2010). The low proteolysis in chloroplast could explain the high yields of recombinant proteins reached in this organelle (Surzycki et al., 2009; Potvin and Zhang, 2010). However, as far as glycosylated proteins such as biopharmaceuticals are concerned, chloroplast lacks the enzymatic machinery required for *N*-glycosylation. Therefore, for biopharmaceutical production, both nuclear expression and protein transport through the secretory pathway of microalgae are required.

Despite recent progress, nuclear expression remains challenging in *C. reinhardtii* (Specht et al., 2010). Indeed, low levels of expression usually observed could result from transgene silencing (Cerutti et al., 1997; Shaver et al., 2010; Rasala et al., 2012). Its GC-rich genome (Merchant et al., 2007) has been also thought to be a hindrance to the expression of foreign genes since it introduces a codon usage bias. Several strategies have been developed during the last decade to circumvent these problems. Among them, the necessity to optimize the coding sequence of the gene of interest appears to be essential for improving the nuclear expression of the foreign protein (Fuhrmann et al., 1999). With regards to promoter sequences, a few assays have been performed to express reporter genes under the control of the plant constitutive CaMV35S promoter (Ruecker et al., 2008; Díaz-Santos et al., 2013) or of other viral sequences (Ruecker et al., 2008). Recently, a high light-inducible promoter from *Dunaliella* has also been investigated in *C. reinhardtii*. This promoter has been shown to drive efficiently the expression of the luciferase reporter gene but it has not been used for expression of any biopharmaceutical yet (Park et al., 2013). Currently, the major part of nuclear transgenes are expressed through a hybrid promoter, resulting from the fusion of the photosystem I complex (PSAD) (Fischer and Rochaix, 2001) or the Ribulose Bisphosphate Carboxylase Small Subunit (RBCS2) (Kindle, 1998) promoters with the HSP70A promoter which allows increasing the expression of the transgene (Schroda et al., 2000). In addition, several regulatory sequences have been included in the transgene coding sequence to enhance its expression. Thus, the insertion of the first intron of RBCS2 (Lumbreras et al., 1998), but also of the second and third introns (Eichler-Stahlberg et al., 2009) are required to increase the efficacy of the HSP70A/RBCS2 promoter. Compared to the CaMV35S promoter, these chimeric promoters remain actually the most efficient to drive the constitutive expression of nuclear transgenes in *C. reinhardtii* (Ruecker et al., 2008; Eichler-Stahlberg et al., 2009; Neupert et al., 2009; Rasala et al., 2012; Kumar et al., 2013). However, with a yield of secreted EPO estimated to 0.1 mg per liter of culture medium (Eichler-Stahlberg et al., 2009), the expression level of EPO under the control of the HSP70A/RBCS2A promoter remains too low for large scale production.

Rasala et al. (2012) developed a new vector in which the gene of interest is fused to the *BLE* gene of selection (which confers the resistance to bleomycin) *via* the nucleotide sequence encoding the foot-and-mouth-disease-virus 2A self-cleavage peptide (FMDV 2A) under the control of the HSP70A/RBCS2 promoter. The resulting translated product is processed into two proteins, with the 2A peptide fused to the C-terminal end of the first protein as already described for other expressions using a 2A self-cleavage peptide (Ho et al., 2013). This tandem expression allows the selection of transformants exhibiting a higher transgene expression. Finally, Neupert et al. (2009) used UV mutagenesis to generate new *C. reinhardtii* strains presenting the advantage to increase the expression of nuclear transgenes. Indeed, using one of these strains (UVM4) transformed with the specific vector pcCAgLUC allowing the expression of the luciferase protein fused to the predicted signal peptide of the extracellular carbonic anhydrase 1 (CAH1), Lauersen and coworkers demonstrated that the amount of secreted recombinant proteins could be significantly improved, reaching up to 10 mg per liter of culture (Lauersen et al., 2013a). However, despite these significant achievements, efforts are still necessary to make *C. reinhardtii* competitive with CHO cell lines, for which yield between 5 and 10 g/L of recombinant protein is currently obtained (Demain and Vaishnav, 2009).

#### Other microalgae

Recently, other microalgae species have also been shown to be of interest as new expression systems. For example, two recent studies reported the expression of a monoclonal human IgG antibody against the Hepatitis B and its respective antigen in the diatom *P. tricornutum*. This antibody was either secreted in the culture medium or expressed in fusion to a DDEL (instead of KDEL which is used as a standard ER retention signal in other eukaryotes) sequence allowing its retention within the endoplasmic reticulum (ER) of *Phaeodactylum tricornutum* (Hempel et al., 2011b; Hempel and Maier, 2012). In contrast to *C. reinhardtii*, the codon usage in *P. tricornutum* is much closer to that of human (Heitzer et al., 2007), which could give advantage to this diatom for the production of biopharmaceuticals. In *P. tricornutum*, nuclear expression of transgenes is usually mediated through the promoter of the *FCPA* gene which encodes the fucoxanthin chlorophyll a/c binding protein (Apt et al., 1996; Zaslavskaia et al., 2000) or through the nitrate reductase promoter which is induced when ammonium is replaced by nitrates as nitrogen source in the culture medium (Poulsen and Kröger, 2005; Gonzalez et al., 2011; Hempel et al., 2011b; Hempel and Maier, 2012; Stork et al., 2012). This specific nitrate reductase inducible promoter was used to express the fully-assembled human IgG antibody against Hepatitis B leading to 1.5–2.5 mg of recombinant antibody per liter of culture medium depending of the different clones (Hempel and Maier, 2012) and 21 mg per gram of algal dry weight for the ER-retained form of the antibody (Hempel et al., 2011b).

Expression of commercially relevant proteins in other species of microalgae remains less reported, since tools for nuclear transformation are still missing. As for *C. reinhardtii*, the CaMV35S promoter has been shown to be efficient in several other green microalgae, including *C. ellipsoidea* (Jarvis and Brown, 1991; Chen et al., 2001; Kim et al., 2002), *C. vulgaris* (Hawkins and Nakamura, 1999), *Haematococcus pluvialis* (Kathiresan et al., 2009) and *D. salina* (Geng et al., 2003; Feng et al., 2009; Chai et al., 2013) but in most of the cases, the expression levels achieved remained low. Some studies reported the use of other foreign promoters to drive the nuclear expression of heterologous proteins in *Chlorella* sp., such as the *C. reinhardtii* RBCS2 promoter which has been used successfully in *Chlorella ellipsoidea* for transient expression of recombinant protein or resistance expression (Hawkins and Nakamura, 1999; Kim et al., 2002). In contrast, the ubiquitin maize promoter seems to be promising in *Chlorella* sp., since it allows the stable expression of a rabbit gene encoding for an α-defensin (Bai et al., 2013). In some cases, the omega element of Tobacco Mosaic Virus (TMV) which is part of the 5′UTR has been used to enhance translation efficiency (Chen et al., 2001), leading to a yield of about 11 mg/L of recombinant protein (Bai et al., 2013). This ubiquitin-Ω promoter was also successfully used in *D. salina* to stably express a gene encoding the hepatitis B surface antigen (Geng et al., 2003).

### Expression of secreted therapeutic recombinant proteins in microalgae

As mentioned previously, most of the recombinant proteins that have been produced in *C. reinhardtii* so far were expressed in the chloroplast because of the high level of protein accumulation reached in this organelle. Thus, among the 20 recombinant proteins of industrial interest expressed in *C. reinhardtii* (for a recent review, see Rasala and Mayfield, 2014), only 3 of them have been expressed successfully through the nuclear genome: a xylanase (Rasala et al., 2012), an ice binding-protein (Lauersen et al., 2013b) and the secreted EPO (Eichler-Stahlberg et al., 2009). As the xylanase and ice binding-protein are not therapeutical proteins, we will not discuss them further in this review. In addition, by using the signal peptide of the *C. reinhardtii* gene *ARS2* encoding a periplasmic arylsulfatase, the EPO has been produced in the culture medium of *C. reinhardtii* (Eichler-Stahlberg et al., 2009; Table 1). Furthermore, as previously indicated, Hempel and Maier (2012) demonstrated the capability of *P. tricornutum* to synthesize and secrete a full length functional human IgG antibody against the Hepatitis B virus surface antigen. This study clearly demonstrates that diatoms are able to produce and correctly assemble complex proteins without affecting their biological activity (Table 1).

**Table 1:** **Post-translational modifications of biopharmaceuticals expressed in the secretory system of microalgae**.

**Expressed protein**	**Promoter**	***N*-glycosylation site^a^**	**Glycosylation^b^**	**References**
***IN C. REINHARDTII***				
Human EPO	Hsp70A/RbcS2	Yes	Yes	Eichler-Stahlberg et al., 2009
***IN P. TRICORNUTUM***				
Monoclonal human IgG against the Hepatitis B surface antigen	Nitrate reductase	Yes	Yes	Hempel et al., 2011b; Hempel and Maier, 2012
Hepatitis B surface antigen	Nitrate reductase	Yes	No	Hempel et al., 2011b
***IN D. SALINA***				
Hepatitis B surface antigen	Maize Ubiquitin + Ω TMV enhancer	Yes	No	Geng et al., 2003

a *Predicted N-glycosylation sites by bio-informatic analysis of the protein sequence*.

b *Experimental evidence of the presence of N-glycans attached to the N-glycosylation site*.

Little information is available on the post-translational modifications acquired by these microalgae-made therapeutic proteins in the secretory pathway. For example, the recombinant EPO expressed in *C. reinhardtii* exhibited a molecular mass of about 33 kDa suggesting the presence of post-translational modifications such as the addition of glycans on the microalgae-made EPO. This is consistent with the fact that the EPO is known to possess 3 *N*-glycosylation and one O-glycosylation sites (Table 1) (Lingg et al., 2012). Moreover, affinoblotting with concanavalin A, a lectin specific for oligomannoside structures (Fitchette et al., 2007), of the ER-resident recombinant IgG expressed in *P. tricornutum* also suggested that this microalgae-made antibody is glycosylated (Table 1) (Hempel et al., 2011b). However, no structural detailed analyses are reported regarding the glycans attached to those microalgae-made biopharmaceuticals. Since the *N*-glycosylation of biopharmaceuticals is critical for their half-life, stability and biological activity (for a recent review, see Lingg et al., 2012), it is therefore essential to characterize the *N*-glycosylation process of secreted protein in microalgae.

## *N*-glycosylation pathways in microalgae

### General aspects of *N*-glycosylation in eukaryotes

**Figure 1:**
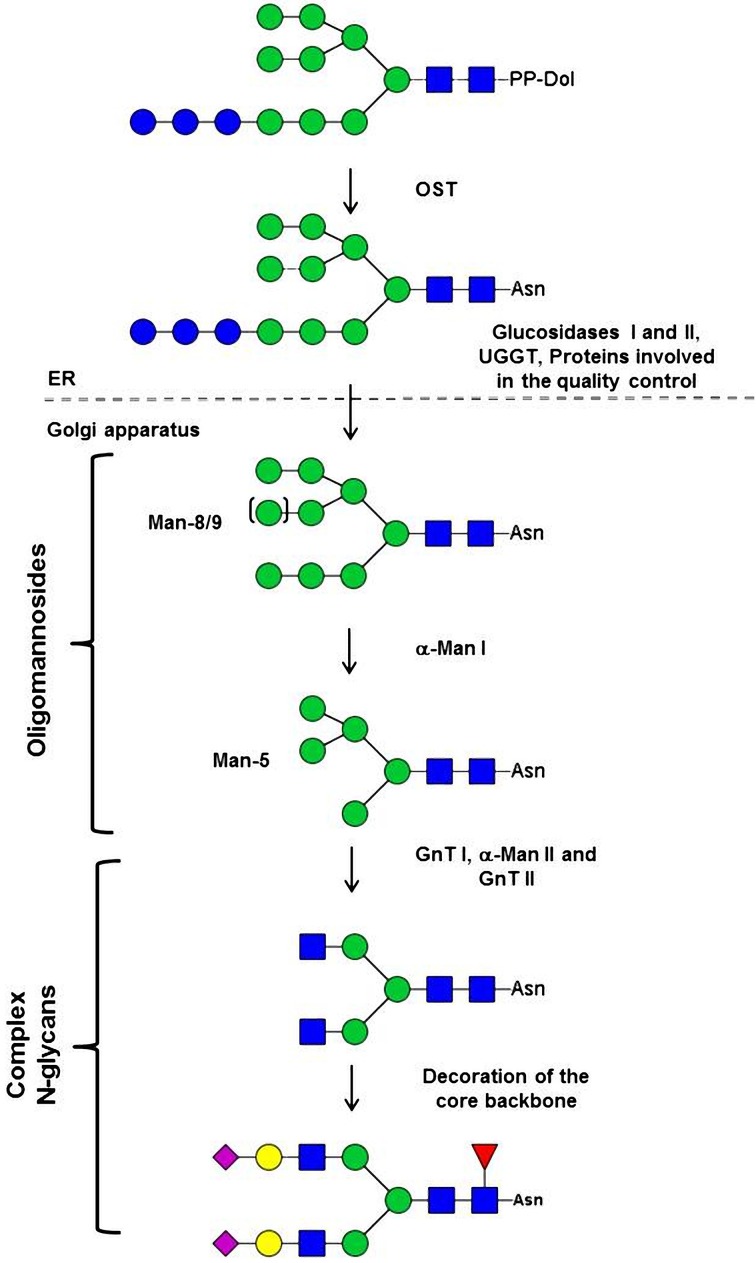
**Mammalian *N*-glycosylation pathway**. This pathway starts in the endoplasmic reticulum (ER) and ends in the Golgi apparatus where the major maturation steps lead to sialylated and fucosylated biantennary protein *N*-linked glycans. The *N*-glycan structures presented here are annotated according to the symbolic nomenclature adopted by the *Consortium for Functional Glycomics* (Varki et al., 2009). PP-Dol: Pyrophosphate Dolichol; OST: oligosaccharyltransferase; Asn, asparagine; UGGT, UDP-glucose glycoprotein glucosyltransferase; α-Man, α-mannosidase; GnT, N-acetylglucosaminyltransferase; Man-8/9, oligomannoside bearing 8/9 mannose residues; Man-5, oligomannoside bearing 5 mannose residues. 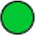 Mannose 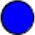 Glucose 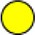 Galactose 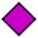 Sialic acid 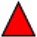 Fucose 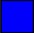 N-acetylglucosamine.

*N*-glycosylation is a major co- and post-translational modification of proteins in eukaryotes occurring in both the ER and the Golgi apparatus (Figure 1). In this process, a lipid-linked oligosaccharide is first assembled by the stepwise addition of monosaccharides on a dolichol pyrophosphate on the cytosolic face and then in the lumen of the ER (Burda and Aebi, 1999). This precursor is then transferred by the oligosaccharyltransferase (OST) complex onto the asparagine residues of the consensus Asn-X-Ser/Thr sequences of a protein (Burda and Aebi, 1999). In 3.5% of the cases, other sequences like Asn-X-Cys, Asn-X-Val have been proven to be glycosylated on endogenous or recombinant protein produced both in mammals or plant cells (Gil et al., 2009; Zielinska et al., 2010; Matsui et al., 2011). The precursor is deglucosylated by the α-glucosidases I and II and then reglucosylated by an UDP-glucose: glycoprotein glucosyltransferase (UGGT) to ensure the proper folding of the nascent protein through its interaction with ER-resident chaperones, such as calnexin and calreticulin. These ER events are conserved in eukaryotes because they are crucial for efficient protein folding and oligomerization (Helenius and Aebi, 2001). In contrast, evolutionary adaptation of *N*-glycan processing in the Golgi apparatus has given rise to a variety of organism-specific complex structures (Varki, 2011). First, α-mannosidases degrade the oligosaccharide precursor into oligomannosides ranging from Man_9_GlcNAc_2_ to Man_5_GlcNAc_2_ (Man-9 to Man-5). *N*-acetylglucosaminyltransferase I (GnT I) then transfers a first *N*-acetylglucosaminyl (GlcNAc) residue on Man-5 and initiates the synthesis of a large variety of structurally different complex-type *N*-glycans. In this GnT I-dependent *N*-glycan maturation, the processing continues by the removal of two mannosyl residues and the transfer of a second terminal GlcNAc residue, thus resulting in the synthesis of a core GlcNAc_2_Man_3_GlcNAc_2_ which are common to mammals and all land plants studied so far (Lerouge et al., 1998; Wilson et al., 2001; Gomord et al., 2010; Varki, 2011) (Figure 1). This core is then decorated by the action of a specific repertoire of glycosyltransferases that differ from one organism to another. As a consequence, mature proteins leaving the secretory pathway carry organism-specific complex *N*-glycans allowing the protein to acquire a set of glycan-mediated biological functions (Varki, 1993; Gagneux and Varki, 1999). For instance, in mammals, most secreted proteins in blood circulation carry biantennary *N*-glycans decorated with α(1,6)-fucose residues and terminal sialic acids that impact either the protein activity or protein half-life (Figure 1; Lingg et al., 2012). In contrast, plant N-glycans are mainly of biantennary complex type N-glycan carrying a core-β(1,2)–xylose; a core α(1,3)-fucose and eventually terminal Lewis a antennae (Lerouge et al., 1998; Wilson et al., 2001).

By comparison with data available in pluricellular eukaryotes, information concerning protein *N*-glycosylation in microalgae remains very limited. A few studies using lectin blot analysis or enzymatic sequencing suggested that proteins secreted by green microalgae carry mainly oligomannosides or complex *N*-glycans having a core xylose residue (Balshüsemann and Jaenicke, 1990; Grunow et al., 1993; Gödel et al., 2000). More recently, a cell wall glycoprotein from the red microalgae *Porphyridium* sp. was found to carry Man-8 and Man-9 oligomannosides containing 6-*O*-methyl mannoses and substituted by one or two xylose residues (Levy-Ontman et al., 2011).

### Structural investigation of glycan *N*-linked to microalgae proteins of *chlamydomonas reinhardtii* and *phaeodactylum tricornutum*

Deeper insights into the structure of glycans *N*-linked to proteins secreted by *C. reinhardtii* (Mathieu-Rivet et al., 2013) and *P. tricornutum* (Baïet et al., 2011) have been recently reported and *N*-glycosylation pathways in these two microalgae dedicated to biotechnology applications have been proposed. These studies combined data resulting from the search for genes encoding putative enzymes involved in the *N*-glycosylation pathway in the genomic databases and data resulting from biochemical analysis of glycan *N*-linked to secreted proteins.

#### Bio-informatic analysis

**Figure 2:**
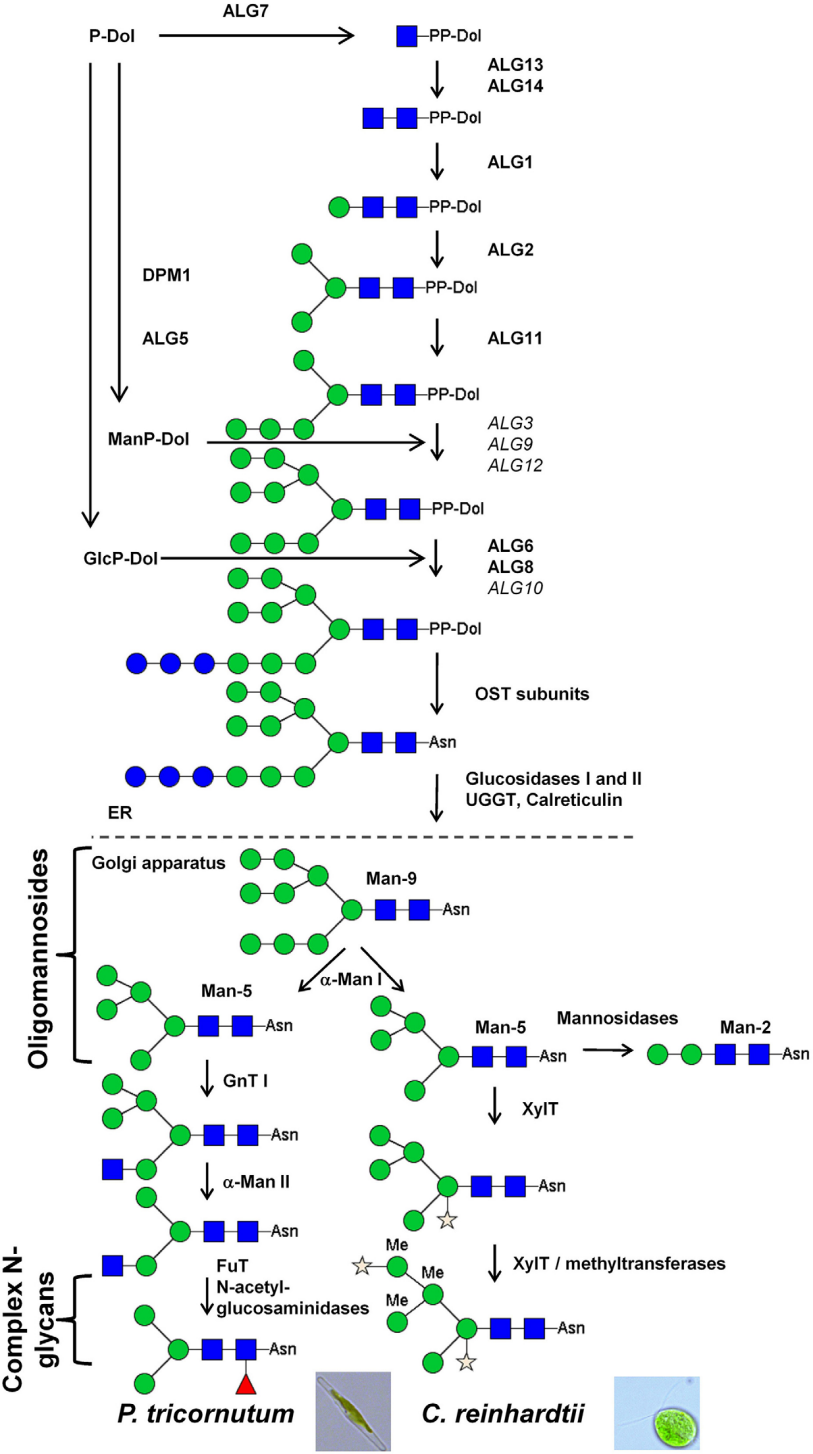
**Comparison of the N-glycosylation pathways in *P. tricornutum* and *C. reinhardtii***. In both microalgae, mature *N*-linked glycans have been structurally characterized. Genomic data are in agreement with the biochemical ones and are summarized in this figure. Sequences predicted in both *C. reinhardtii* and *P. tricornutum* genomes are written in bold. *ALG10* in *P. tricornutum* genome and *ALG3, 6* and *12* in *C. reinhardtii* genome have not been identified yet. The *N*-glycan structures presented in this figure are annotated according to the symbolic nomenclature adopted by the *Consortium for Functional Glycomics* (Varki et al., 2009). DPM1: Dolichol-phosphate mannosyltransferase; ALG: Asparagine-Linked Glycosylation; PP-Dol: Pyrophosphate Dolichol; P-Dol: Dolichol phosphate; OST: oligosaccharyltransferase; Asn, asparagine; UGGT, UDP-glucose glycoprotein glucosyltransferase; GnT, N-acetylglucosaminyltransferase; α-Man, α-Mannosidase; FuT, Fucosyltransferase; XylT, xylosyltransferase; Man-9, oligomannoside bearing 9 mannose residues; Man-5, oligomannoside bearing 5 mannose residue; Man-2, *N*-glycans bearing 2 mannose residues. 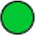 Mannose 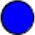 Glucose 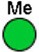 Methyl mannose 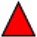 Fucose 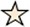 Xylose 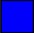 N-acetylglucosamine.

In *C. reinhardtii* and *P. tricornutum* genomes, most of the genes encoding enzymes involved in the biosynthesis of the dolichol pyrophosphate-linked oligosaccharide on the cytosolic face and in the lumen of the ER, named Asparagine-Linked Glycosylation (ALG) (Weerapana and Imperiali, 2006), are predicted (Figure 2). Although some ALG were not clearly identified in the genomes, large oligomannosides up to Man-9 were found in both *C. reinhardtii* (Mathieu-Rivet et al., 2013) and *P. tricornutum* (Baïet et al., 2011) suggesting that the synthesis of the oligosaccharide precursor is similar to the one described in other eukaryotes. In addition to ALG, genes encoding subunits of the oligosaccharyltransferase were also identified in both genomes (Figure 2). Glucosidases I and II, as well as ER-resident UGGT and chaperones such as calreticulin, are also predicted. These proteins are key elements of the quality control of proteins occurring in the ER and are crucial for acquisition of the proper folding of the nascent glycoprotein (Figure 2).

After the transfer of the glycoprotein into the Golgi apparatus, maturation of *N*-linked glycans starts with the trimming of Man-9/-8 by α-mannosidases I (α-Man I) that generate oligomannosides ranging from Man-8 to Man-5. Genes encoding α-Man I are predicted in *C. reinhardtii* and *P. tricornutum* genomes (Figure 2) suggesting that Golgi mediated trimming of oligommanosides also occurs in both microalgae. In higher eukaryotes including animals, insects and land plants, *N*-glycans are then processed by GnT I that transfers a first *N*-acetylglucosaminyl (GlcNAc) residue on the α(1,3)-mannose arm of Man-5 (Figure 1). Thus, GnT I is a key enzyme since it is the first glycosyltransferase occurring in the Golgi apparatus in the GnT I-dependent pathway giving rise to complex *N*-glycans that are required for normal morphogenesis in pluricellular organisms (Ioffe and Stanley, 1994; Metzler et al., 1994). A sequence encoding for a GnT I is predicted in *P. tricornutum* (Baïet et al., 2011) but not in *C. reinhardtii* (Mathieu-Rivet et al., 2013) suggesting that *N*-linked glycans from these two microalgae could be processed in the Golgi apparatus according to two different pathways, referred to as GnT I-dependent and GnT I-independent pathways (Zhu et al., 2004; Crispin et al., 2006; Grass et al., 2011).

In both GnT I-dependent and independent pathways, the next steps mainly consist in the transfer of monosaccharides on the *N*-glycan core to synthesize highly diverse complex *N*-glycans. Among residues that are added to the core, fucosyl residues either α(1,3)- or α(1,6)-linked to the proximal GlcNAc are commonly observed. In both microalgae, sequences encoding for putative α(1,3)-fucosyltransferases were identified in the genomes (Baïet et al., 2011; Mathieu-Rivet et al., 2013). Search for other genes encoding for putative *N*-glycan processing enzymes revealed sequences exhibiting homologies with α-Man II and GnT II in *P. tricornutum* but functional characterizations remain necessary to assess their enzymatic activities.

#### Biochemical analysis

A detailed glycomic analysis of *C. reinhardtii* proteins indicated that both secreted and membrane-bound proteins carry oligommanosides ranging from Man-2 to Man-5 and also complex *N*-glycans containing 6-*O*-methyl mannoses and substituted by one or two xylose residues (Figure 2) (Mathieu-Rivet et al., 2013). Similar complex structures have been previously found in a cell wall glycoprotein isolated from the red microalgae *Porphyridium sp*. (Levy-Ontman et al., 2011). However, the location of the xylose residue on the core *N*-glycan differs in these two microalgae, since it is described to be linked to the chitobiose unit in *Porphyridium* rather than to C2 of the β-mannose as described in *C. reinhardtii* and as previously reported in land plants (Figure 2) (Lerouge et al., 1998). In agreement with bio-informatics data suggesting the absence of GnT I, these complex *N*-glycans are likely to result from the transfer of xylose residues onto Man-5 oligomannosides in a GnT I-independent manner. Then, methylation of mannose residues is thought to occur after xylosylation of complex *N*-glycans in *C. reinhardtii* as proposed in Figure 2. Alternatively, Man-5 can be trimmed by mannosidases down to Man-2 (Figure 2). Although *O*-methyl mannose residues was previously reported in some eukaryotes (Staudacher, 2012), complex *N*-glycans from *C. reinhardtii* highly differ from glycans described in animals and land plants. We postulate that xylosylation and methylation of oligomannosides in these microalgae may protect glycoproteins against deglycosylating enzymes such as endoglycosidases or peptide *N*-glycosidases.

In contrast to complex *N*-glycans from *C. reinhardtii*, glycans *N*-linked to proteins secreted by the diatom *P. tricornutum* can be processed through a GnT I-dependent pathway into partially fucosylated Man-3 (Figure 2) (Baïet et al., 2011). The GnT I gene predicted in the *P. tricornutum* genome was demonstrated to encode an active enzyme able to restore the maturation of *N*-linked glycans into complex-type *N*-glycans in the CHO *Lec1* mutant, defective in its endogenous GnT I (Baïet et al., 2011). The authors have proposed that this fucosylated *N*-linked glycan results from the addition of a terminal GlcNAc residue by GnT I on Man-5 followed by removal of two Man residues by an α-Man II and the transfer of a fucose residue by the predicted α(1,3)-FuT (Baïet et al., 2011). As illustrated in the proposed pathway depicted in Figure 2, the terminal GlcNAc introduced in the Golgi apparatus by the *P. tricornutum* GnT I is then likely removed in the secretory pathway by glucosaminidases as previously described in land plants and insect (Vitale and Chrispeels, 1984; Altmann et al., 1995). Efficient complementation of the CHO *Lec1* mutant is the main argument supporting that this diatom processes its *N*-glycans through a GnT I-dependent pathway. This result also indicates that the microalgae transferase is properly targeted to the Golgi apparatus when expressed in mammalian cells.

In these two studies performed on *P. tricornutum* and *C. reinhardtii N*-glycan processing and as described for land plants (Séveno et al., 2004; Zeleny et al., 2006), no sialic acid residues were identified (Baïet et al., 2011; Mathieu-Rivet et al., 2013). Sialic acids are terminal residues of O- and *N*-glycans that are specifically involved in many biological functions in mammals, such as the half-life of blood proteins and cell-cell adhesion processes (Figure 1) (Varki, 1993; Gagneux and Varki, 1999).

## Glycan-remodeling strategies in microalgae: perspectives

Differences in specificity of Golgi transferases and glycosidases between eukaryotes give rise to glycosylation profiles that differ between mammals and other eukaryotic host cells used as cell factories. As a consequence, glycans *N*-linked to recombinant proteins produced in plants, yeast or even in animal cells differ from the original therapeutic proteins. This may result in either a decrease or absence of biological activity. Furthermore, unsuitable *N*-glycan structures introduced by the expression system can induce immune responses in humans and generate adverse reactions (Van Beers and Bardor, 2012), as reported for α(1,3)-Gal epitope or Neu5Gc on therapeutic drugs (Chung et al., 2008; Padler-Karavani et al., 2008). Similarly, plant synthesize *N*-glycans carrying immunogenic core-xylose and core-α(1,3)-fucose that may induce immune responses in human treated with plant-made biopharmaceuticals (Bardor et al., 2003). In consequence, whatever the expression system that is considered, strategies have been carried out for the *in vivo* remodeling of the protein *N*-glycans in order to obtain structures that meet pharmaceutical requirements. For instance, knock-out of endogenous genes involved in the transfer of core immunogenic epitopes have been carried out to engineer oligosaccharides synthesized by plant cell into human-compatible structures (Cox et al., 2006; Schähs et al., 2007; Strasser et al., 2008). Furthermore, knock-in methodologies based on the expression in the host cells of mammalian enzyme have been developed to *in vivo* introduce missing glycan sequences. In plants, these efforts resulted in the production of plant-derived therapeutic proteins carrying *N*-glycans similar to those found on human counterpart (Palacpac et al., 1999; Bakker et al., 2001, 2006; Misaki et al., 2006; Paccalet et al., 2007; Rouwendal et al., 2007; Castilho et al., 2008, 2010, 2013).

As reported in section Microalgae as Alternative Systems for Production of Recombinant Proteins, *C. reinhardtii* and *P. tricornutum* have been evaluated for their capacity to express therapeutic proteins. The recent characterization of glycans *N*-linked to their secreted proteins allows the design of strategies to engineer their *N*-glycan pathways for the production of human-compatible therapeutic proteins.

### Microalgae for the production of lysosomal proteins

**Figure 3:**
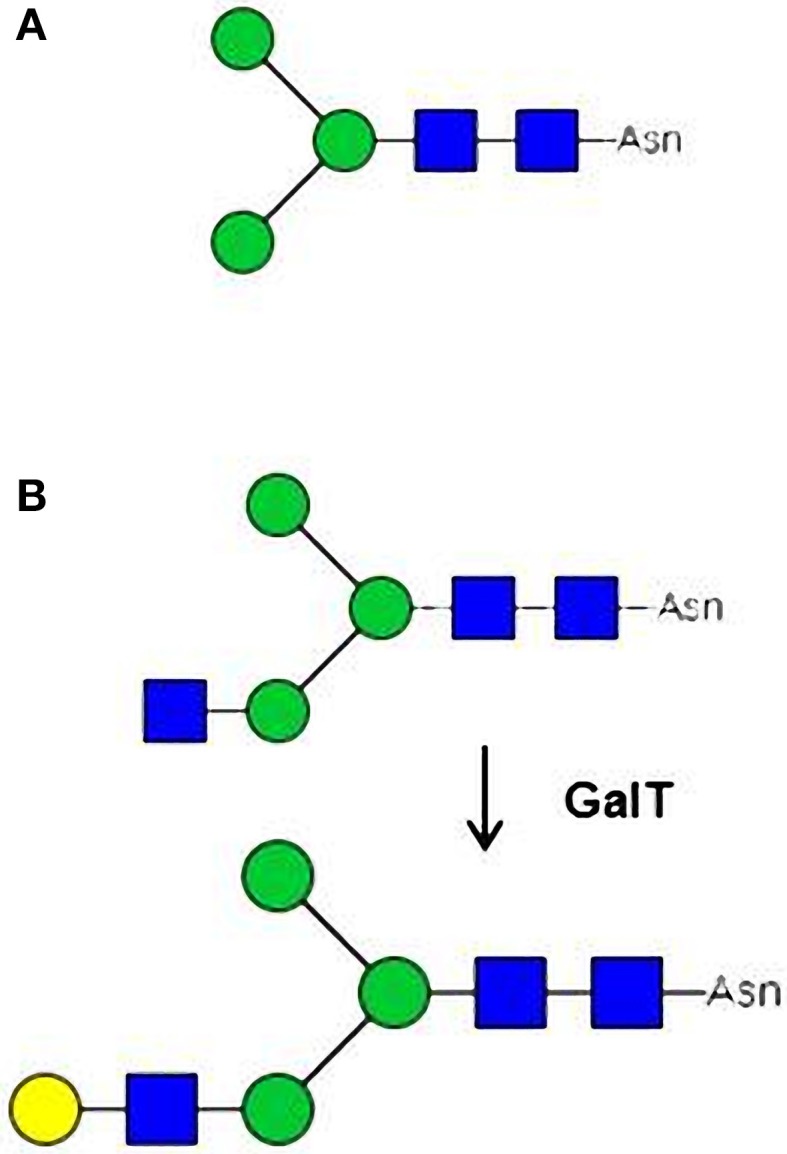
**Engineering of the *N*-linked glycans associated to biopharmaceuticals. (A)** Man-3 oligomannoside N-linked to the recombinant glucocerebrosidase used for Enzyme Replacement Therapy in patients suffering from Gaucher's disease, a lysosomal storage disease. **(B)** Trapping in the Golgi apparatus of intermediate GlcNAc-terminated N-glycans by expression of a mammalian β(1,4)-galactosyltransferase (GalT). 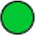 Mannose 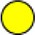 Galactose 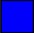 N-acetylglucosamine.

Although complex *N*-glycans have been identified on proteins secreted by *C. reinhardtii* and *P. tricornutum*, most abundant oligosaccharides *N*-linked to proteins are oligomannosides ranging from Man-2 to Man-5 in *C. reinhardtii* (Mathieu-Rivet et al., 2013) and from Man-5 to Man-9 in *P. tricornutum* (Baïet et al., 2011) (Figure 2). These *N*-linked glycans are appropriate for the production of lysosomal therapeutics. For example, glucocerebrosidase is a glycoprotein drug administered intravenously into patients suffering from Gaucher's disease, a lysosomal storage disease. The effective targeting and internalization of this therapeutic drug into macrophages depend on terminal mannose residues of its *N*-glycans which are recognized by macrophage cell surface mannose receptors (Van Patten et al., 2007). For treatment, exogenous glucocerebrosidase is administered intravenously into patients. The preparation of the current glucocerebrosidase expressed in CHO cells (Cerezyme®) requires *in vitro* post-purification and exoglycosidase digestions to expose the trimannose core (Man_3_GlcNAc_2_, Man-3) using a combination of at least three enzymes namely sialidase, galactosidase and *N*-acetylglucosaminidase (Figure 3A). These *in vitro* glyco-engineering steps increase considerably the production costs (Weinreb, 2008). Therefore, alternative expression systems that are capable of producing mannose terminated *N*-glycans have been developed as safe and cost effective production methods. These include cultured carrot cells (Shaaltiel et al., 2007) and *A. thaliana cgl* transgenic plants (He et al., 2012). However, the analysis of the *N*-glycan profiles of these glucocerebrosidase bio-similars revealed Man-5 with variable amount of Man-6 to Man-9 structures (for example low amount of Man 6 to Man-8 on glucocerebrosidase expressed in the Arabidopsis *cgl* mutant). The presence of such structures required careful biosafety studies to evaluate the possible binding to serum mannose binding lectin (MBL) and immunogenicity (Van Patten et al., 2007; Grabowski et al., 2014). The results described for *C. reinhardtii* (Mathieu-Rivet et al., 2013) suggest that this green microalgae could become in the future an appropriate platform for the production of a glucocerebrosidase bio-better which would naturally carry Man-2 to Man-5 glycan structures without any *in vitro* trimming of mannose residues (Figure 2).

### Microalgae for the production of proteins carrying human-compatible complex-type *N*-glycans

Based on data reported in section N-glycosylation Pathways in Microalgae and Figure 2, the production in microalgae of therapeutic proteins carrying human-like complex glycans will require intensive remodeling of their glycan pathways. Although no information is available on their immunogenicity, glycan epitopes identified on microalgae complex *N*-glycans will likely induce adverse effects in humans. Removal of these putative immunogenic glyco-epitopes, such as xylose and fucose residues, as well as methyl groups on mannose residues, will first require to identify gene encoding the corresponding glycosyltransferases and methyltransferases and then to carry out appropriate knock-out strategies such as RNA interference; artificial microRNA which have already been developed in microalgae (Zhao et al., 2009; Cerutti et al., 2011). If putative FuT genes have already been identified because of their high homologies with plant α(1,3)-FuT, the identification of sequences coding for xylosyltransferases (XylT) will be more tricky. Only the β (1,2)-XylT family involved in complex *N*-glycan biosynthesis in land plants have been biochemically characterized so far (Strasser et al., 2000). To date, no specific domain for XylT activity has been clearly identified and so, search by homology for XylT in microalgae genomes failed to clearly identify putative candidates.

As previously reported in land plants, “missing” residues on glycan *N*-linked to therapeutic proteins can be introduced by co-expression with appropriate glycosyltransferases. Results regarding the complementation of CHO *Lec1* mutant by *P. tricornutum* GnT I (Baïet et al., 2011) showed that the targeting of Golgi glycosyltransferases is conserved between microalgae and higher eukaryotes. As a consequence, we can expect that the expression in microalgae of heterologous glycosyltransferases would allow the *in vivo* remodeling of their *N*-glycan pathways. This heterologous expression of glycosyltransferases should be particularly suitable for the remodeling of *N*-glycans in *P. tricornutum* because this diatom possesses a functional GnT I making its *N*-glycosylation pathway closer to the mammalian one (Figures 1, 2). The terminal GlcNAc introduced by GnT I constitute the starting point for the building of antennae in mammalian complex *N*-glycans (Figure 1). We postulated that in *P. tricornutum* this terminal GlcNAc is lost during the trafficking of the glycoprotein by action of N-acetylglucosaminidases (Figure 2). However, the expression of a mammalian β (1,4)-galactosyltransferase in *P. tricornutum* would allow the transfer in the Golgi apparatus of a terminal Gal on the terminal GlcNAc, thus giving rise to a Galβ (1,4)GlcNAc extension that will protect this N-acetylglucosamine residue introduced by GnT I from degradation by N-acetylglucosaminidases downstream in the secretory pathway (Figure 3B). In addition, this extension corresponds to glycosidic motif found on some therapeutic proteins such as IgG. This strategy based on the trapping of intermediate GlcNAc-terminated *N*-glycans has already been successfully carried out in land plants (Bakker et al., 2001; Huether et al., 2005; Vézina et al., 2009) and insect cells (Hollister et al., 1998).

### Search for microalgae having appropriate *N*-glycosylation pathways

As *in vivo* engineering of the *N*-glycosylation pathways in *C. reinhardtii* and *P. tricornutum* may represent a tricky work, exploration for a microalga that exhibits a more appropriate protein glycosylation has to be considered. A first overview of protein *N*-glycosylation in various microalgae species can be draw on the basis of public genomic databases. In addition to *C. reinhardtii* and *P. tricornutum*, more than 15 genomes from other microalgae are now available (e.g., *Thalassossira pseudonana*; *Cyanidioschyzon merolae*; *Ostreococcus tauri* and *Ostreococcus lucimarinus*; *Micromonas pusilla*; *Chlorella vulgaris*; *Coccomyxa subellipsoidea*; *Nannochloropsis gaditana*; *Monoraphidium neglectum*; Table 2) (Ambrust et al., 2004; Misumi et al., 2005; Palenik et al., 2007; Worden et al., 2009; Blanc et al., 2010, 2012; Radakovits et al., 2012; Vieler et al., 2012; Bogen et al., 2013). Searches for ALG genes and other sequences encoding proteins involved in the ER protein quality control indicated that enzymes of the ER machinery are predicted in all genomes suggesting that ER steps of the *N*-glycan pathway are conserved over the microalgae phyla as discussed recently in Levy-Ontman et al. (2014). With regard to Golgi events, Table 2 summarizes and reports the genes predicted to encode enzymes involved in the maturation of the *N*-glycans. As observed for *C. reinhardtii* and *P. tricornutum*, α-mannosidases (CAZy GH 47) and α(1,3)-fucosyltransferases (CAZy GT10) are predicted in most microalgae genomes. In contrast, genes encoding GnT I are not predicted in all genomes (Table 2; Baïet et al., 2011). For instance, as for *C. reinhardtii*, no GnT I was found in *Volvox* and *Ostreococcus* species whereas this key transferase is predicted in most other microalgae, including haptophytes and cryptophytes (Table 2; Baïet et al., 2011). As demonstrated in *C. reinhardtii* and *P. tricornutum*, this indicates that the Golgi maturation of protein *N*-linked glycans into complex oligosaccharides could either occur through a GnT I-dependent pathway or a GnT I-independent pathway in microalgae. In the context of microalgae-made biopharmaceutical production, this feature must be considered since the engineering of microalgae *N*-glycans into human compatible oligosaccharides would be facilitated if a GnT I-dependent machinery already exists in the strains selected as an expression system.

**Table 2:**
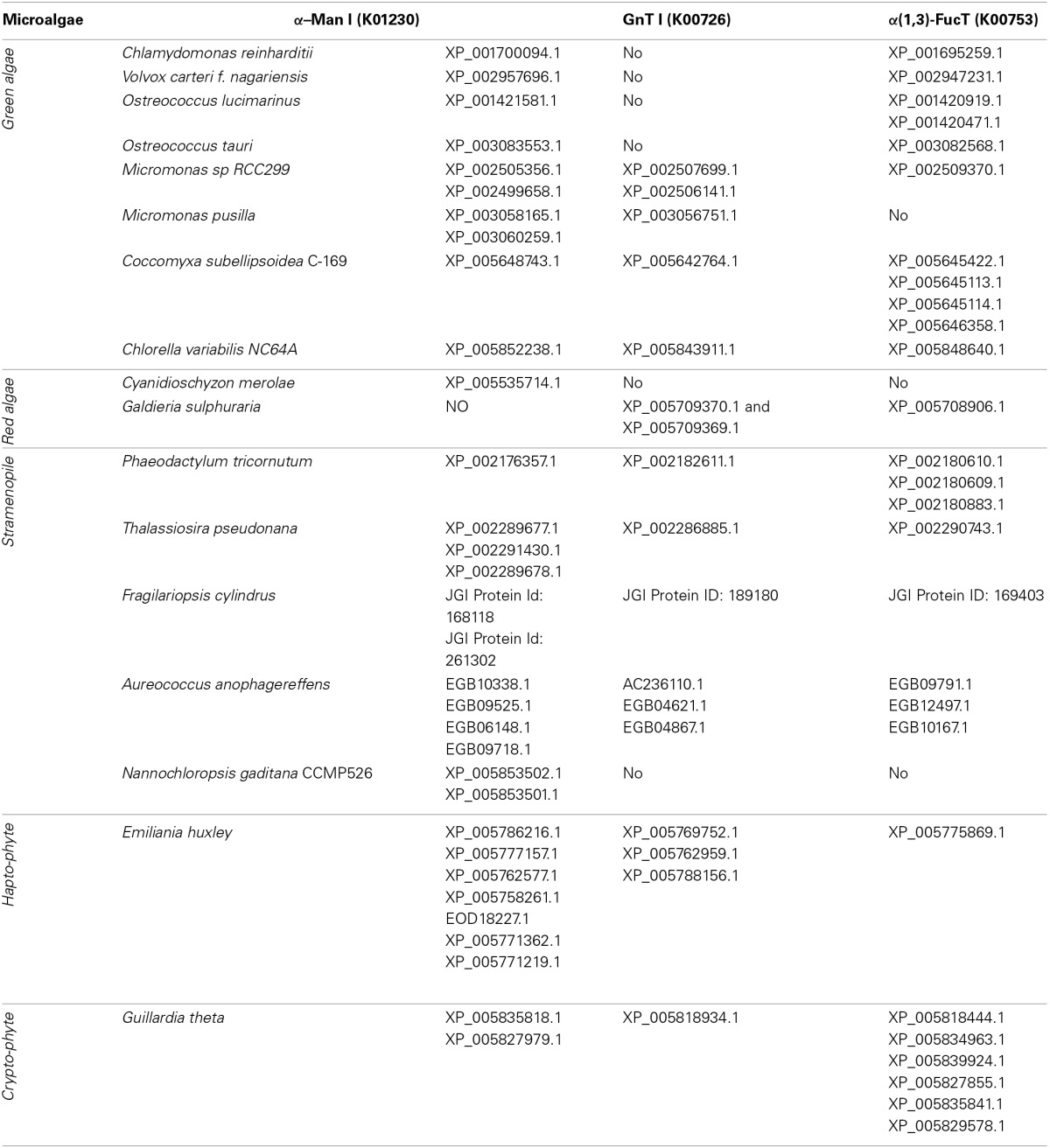
**Genes predicted in microalgae genomes encoding proteins involved in *N*-glycan Golgi maturation such as α-Man I, mannosyl oligosaccharide alpha-1,2-mannosidase; GnT I, alpha-1,3-mannosyl-glycoprotein beta-1,2-N-acetylglucosaminyltransferase; α (1,3)-FucT, core α (1,3)-fucosyltransferase**.

*Sequences were retrieved using the KEGG orthology or by search with the protein's name (as defined in KEGG) in JGI, Phytozome or Genome at NCBI databanks. In addition, search for candidates was carried out by BLASTP or TBLASTN using human alpha-1,2-mannosidase protein sequences, rabbit GnTI (P27115) and Arabidopsis thaliana α(1,3)-FucT (Q9LJK1 and Q9FX97) as query sequences*.

## Conclusions

In conclusion, it can be considered that remodeling *N*-glycans as depicted in Figure 2 into human-compatible oligosaccharides would be a difficult challenge to attend. However, recent production in plants of therapeutic proteins carrying sialylated biantennary *N*-glycans demonstrates that such a glycan engineering can be achieved in a plant expression system (Castilho et al., 2013). It should also be noticed that only a few microalgae species have been investigated and so, data reported in this review should be considered as a starting point regarding protein glycosylation mechanisms occurring in these unicellular organisms and will serve as foundation for further glycobiology works. Efforts have now to be carried out to get more information concerning protein N-glycan structures and their pathways in different phyla to identify microalgae species that are more appropriate for glycan remodeling strategies into human-like structures. This will also necessitate the characterization of the specificity of Golgi glycosyltransferases, such as α-mannosidases and α(1,3)-fucosyltransferases predicted in the microalgae genomes, to evaluate the efficiency in microalgae of heterologous expression of glycosyltransferases as well as to identify genes encoding transferases (methyltransferases and xylosyltransferases) involved in the synthesis of putative immunogenic epitopes with the final goal to selectively inactivate their expression.

## Author contribution

Elodie Mathieu-Rivet and Marie-Christine Kiefer-Meyer equally contributed to this work by drafting the manuscript and performing the genomic analyses of the microalgae genomes which are summarized in the Table 2 of the manuscript. Gaëtan Vanier, Clément Ovide and Carole Burel participated to the writing and took care of the figure drawing. Patrice Lerouge and Muriel Bardor came out with the idea of this review, participate to the writing and coordinated the efforts from the team prior to take care of the submission of the manuscript.

### Conflict of interest statement

The authors declare that the research was conducted in the absence of any commercial or financial relationships that could be construed as a potential conflict of interest.
